# Evaluation of an Italian *Phlebotomus perfiliewi* (Larroussius) wild population as a permissive vector species for *Leishmania tropica* and *L. major* transmission

**DOI:** 10.1186/s13071-025-07170-7

**Published:** 2025-12-05

**Authors:** Ilaria Bernardini, Claudia Mangiapelo, Eleonora Fiorentino, Stefania Orsini, Riccardo Bianchi, Anna Rosa Sannella, Aldo Scalone, Trentina Di Muccio, Gioia Bongiorno

**Affiliations:** 1 Department of Infectious Diseases, Unit of Vector-Borne Diseases, Istituto Superiore Di Sanità Rome, Rome, Italy; 2 Department of Public Health, Experimental and Forensic Medicine, University of Pavia, Pavia, Italy

**Keywords:** *Phlebotomus perfiliewi*, *Leishmania tropica*, *Leishmania major*, Experimental infection, Permissive species

## Abstract

**Background:**

Among pathogenic *Leishmania* species, *L. major* and *L. tropica*, occur mainly in North African and Middle Eastern countries, primarily transmitted by *Phlebotomus papatasi* and *P. sergenti* respectively. In Italy, *P. perfiliewi* is the second most abundant sand fly species involved in *L. infantum* transmission exhibiting opportunistic behaviour in rural and semi-urban settings. Experimental infections were conducted to assess the susceptibility of a wild Italian *P. perfiliewi* population to the development of *L. tropica* and *L. major*.

**Methods:**

Experiments were performed with a wild *P. perfiliewi* population, collected in Magliano in Toscana (Tuscany, Italy). In separate experiments, females were artificially infected with a suspension of *L. tropica* or *L. major* (10^6^ promastigotes/ml). Infection progression was monitored through microscopic midgut dissection and supported by molecular analyses.

**Results:**

Experimental infections with *L. tropica* yielded 5923 engorged females, with an average feeding rate of 25.7% and a survival rate of 70%. The infection positivity rate was 25.2%, significantly lower than that observed in the control species. Nonetheless, parasite migration to the stomodeal valve suggests that *P. perfiliewi* may support the full developmental cycle of *L. tropica*, although less efficiently than the control species. Infections with *L. major* showed a significantly lower mean feeding rate (*p* = 0.05) and a positivity rate of 46.6%, compared to 70.5% in *P. perniciosus* (*p* = 0.03). Still, *P. perfiliewi* appeared capable of supporting *L. major* metacyclogenesis, even under light to moderate parasite loads (*p* = 0.007).

**Conclusions:**

These findings suggest that *P. perfiliewi* can support the development of both *L. tropica* and *L. major* under experimental conditions, with greater susceptibility observed for *L. tropica*. However, without in vivo confirmation or xenodiagnosis, its capacity for natural transmission remains unestablished. Nonetheless, implementing integrated One Health surveillance strategies would be advisable to monitor potential changes in the epidemiology of both exotic and endemic *Leishmania* species.

**Graphical Abstract:**

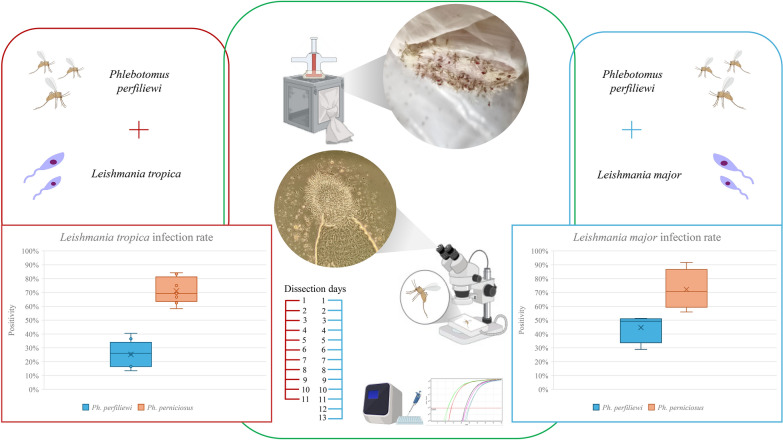

**Supplementary Information:**

The online version contains supplementary material available at 10.1186/s13071-025-07170-7.

## Background

Leishmaniasis, classified as a neglected tropical disease by the World Health Organization (WHO), is traditionally widespread in tropical, subtropical and Mediterranean regions, affecting 98 countries across Africa, Asia, America and Europe, with an estimated 700,000 to 1 million new cases occurring annually [1], affecting both humans and animals. Two main clinical forms are prevalent worldwide: visceral leishmaniasis (VL), a life-threatening condition fatal if untreated, and cutaneous leishmaniasis (CL), a benign but disfiguring skin condition. At least 20 *Leishmania* species (Kinetoplastida, Trypanosomatidae) are pathogenic for humans transmitted by the bite of infected sand flies belonging to the *Phlebotomus* genus. Among over 800 known sand fly species, fewer than 100 have been confirmed or are suspected to transmit human leishmaniasis, with *Phlebotomus* and *Lutzomyia* species acting as vectors in the Old World and the New World, respectively. [2]. Within WHO European Regions, including both the European Union and the Eastern Mediterranean Region, the estimated incidence rates of VL and CL are approximately 1100–1900 and 10,000–17,000 cases per 100,000 populations, respectively [3]. In this areas, leishmaniasis is regarded as an emerging and re-emerging disease, driven by a combination of physiological, epidemiological, and ecological factors, including human immunosuppression, increased human and animal migrations, and climatic and environmental changes, all of which influencing both sand fly distribution and vectorial capacity. [4, 5]. The spatial distribution of *Leishmania* spp. varies considerably: *L. donovani s.s.*, the causative agent of VL, has been reported only sporadically in certain areas of Cyprus and Turkey [6, 7]. *Leishmania infantum*, responsible for the zoonotic forms of VL and CL (ZVL and ZCL), is widespread throughout these countries, with several species of the subgenus *Larroussius* recognized as proven vectors: *P. ariasi*, *P. balcanicus*, *P. kandelakii*, *P. langeroni*, *P. neglectus*, *P. perfiliewi*, *P. perniciosus* and *P. tobbi* [8, 9]. In contrast, *L. major* and *L. tropica* causing CL are present only in countries of North Africa, the Middle East and parts of the Caucasus [4, 9]. The biological cycle of zoonotic* L. major* is maintained primarily by wild rodents, belonging to the genera Psammomys, Meriones, Nesokia, and Rhombomys, which act as reservoir hosts, and by *P. papatasi*, the principal sand fly vector species, widely distributed in endemic regions where human infection also occurs [9]. *L. major* DNA has also been molecularly detected in *P. perfiliewi* [10]. Anthroponotic *L. tropica* on the other hand involves only humans as the only proven reservoir and is mainly transmitted by *P. sergenti s.l.*. Specifically, *P. sergenti* s.s. is the confirmed vector in European regions, but other members of the complex may play a role elsewhere, such as *P. arabicus* in northern Israel, *P. guggisbergi* and *P. aculeatus* in Kenya and *P. saevus* in Ethiopia. Additionally, *P. perfiliewi transcaucasicus, Phlebotomus (Adlerius) sp.and Sergentomyia dentata* have been found molecularly positive for *L. tropica* in Iran [11–15]. All *Larroussius* species tested under laboratory conditions so far have proven to be permissive vectors [16], supporting the development of several *Leishmania* species such as *L. tropica* by *P. perniciosus* and *P. tobbi* [17–19]. In Italy, *P. perfiliewi* is the second most abundant sand fly species widely distributed across northern, central and southern regions, with high density population hotspots. This vector species is associated with anthropized rural and semi-natural environments, showing a high zoophilic aptitude, particularly towards livestock [20]. Although its involvement in *L. infantum* transmission is well known, limited information is available on its broader biology and ecology [21–23]. In Tuscany, a region historically endemic for leishmaniasis [24–27], recent increases in human cases have been reported [28]. Entomological surveys have shown annual peaks in sand fly density [29], however, due to the long incubation period of human leishmaniasis and the often-unnoticed nature of sand fly bites, data on natural phlebotomine infection in leishmaniasis foci may be delayed and underestimated. In the Grosseto Province, *P. perfiliewi* has shown abundant yet uneven distribution over the years, consistent with its ecological preferences, aligned with ecological niches, and *L. infantum* circulation has been confirmed by molecular analysis [30, 31]. Climate change is expected to influence both the distribution of sand fly populations and their vectorial capacity, potentially affecting the dynamics of phlebotomine-borne disease transmission. A key objective, therefore, is to investigate whether local sand fly populations can support the development of “exotic” or non-autochthonous *Leishmania* species that might be introduced through imported cases. In this context, experimental infections were performed to evaluate the susceptibility of a wild Italian *P. perfiliewi* population to *L. tropica* and *L. major* development.

## Methods

Experimental infections were performed to assess the susceptibility of wild *P. perfiliewi* females to *L. tropica* and *L. major*. Infection development and progression was monitored through microscopic examination of dissected midguts, supported by molecular analyses.

### Sand fly maintenance and parasite cultures

Wild *Phlebotomus perfiliewi* females were collected during the summers of 2022–2024 in Magliano in Toscana (Tuscany, Italy). Laboratory-reared *P. perniciosus* (50 individuals per replicate) from an Italian strain served as control. All sand flies were maintained under standardized conditions (≈26 °C, 60–70% humidity, 30% sucrose diet, 17:7 h light:dark) at the Istituto Superiore di Sanità (ISS) (Rome, Italy). Two *Leishmania *strains, isolated from clinical cases and preserved in the CryoBank at ISS, were used for infection. The *L. tropica* strain (MHOM/IT/2016/ISS3183) originated from an Afghan refugee skin lesion, and *L. major *strain (MHOM/IT/2002/ISS2342) obtained from an Italian tourist infected in Morocco. Both strains were confirmed using PCR–RFLP of the ITS1 region and Hsp70 gene sequencing [33].

### Infection test procedure

Promastigotes were cultured minimaizing in vitro passages to maintain infectivity, and amplified in Schneider’s Medium. Parasites were suspended in heat-inactivated rabbit blood at 1 × 10^6^ promastigotes/mL concentration. Blood-unfed or visibly non-gravid sand flies were selected and allowed to feed through an artificial membrane system in the dark for 2 h (see Additional Figure S1), under Biosafety Level 2 (BSL-2). Engorged females were individually transferred to vials containing sucrose solution and a paper substrate to minimize post-feeding mortality. For *L. tropica* infections five replicates were performed in 2022 and three in 2023, while for *L. major* four replicates were conducted in 2024.

### Dissection and microscopic analysis

From 0 to 13 days post blood meal (PBM), up to 10 engorged females per day were dissected. Midgut examination assessed: (i) parasite load (negative: 0, light: 1–100, moderate: 101–1000, heavy: > 1000/gut), (ii) promastigote morphotype (procyclic, nectomonad, leptomonad, metacyclic), and (iii) localization within hindgut, midgut, or stomodeal valve. All wild females were identified to species level using standard entomological keys.

### Molecular analysis

Individual midguts were collected in PBS, centrifuged, and DNA extracted using the Maxwell^®^ 16 Cell DNA Purification Kit (Promega, USA). Parasite load was quantified by SYBR Green quantitative PCR (qPCR) targeting kinetoplast (*k*) DNA [35], using primers described by Mary et al. [35] (forward primer 5ʹ-CTTTTCTGGTCCTCCGGGTAGG-3ʹ and reverse primer 5ʹ-CCACCCGGCCCTATTTTACACCAA-3ʹ) in Bio-Rad iCycler and iQ Real-Time PCR Systems, performed in triplicate under the following cycling conditions: initial denaturation at 95 °C for 3 min, followed by 45 cycles of 95 °C for 10 s, 56 °C for 10 s, and 72 °C for 10 s. Specificity was confirmed by melting curve analysis. Parasite counts were calculated using a standard curve generated from 10-fold serial dilutions of *Leishmania* DNA, mixed with DNA from uninfected sand flies (from 10^6^ to 0.01 parasites per reaction), and multiplied by 50 to estimate load per gut included in each run. Negative controls included sterile water and DNA from *P. perniciosus* males. Positive samples were further genotyped by ITS1-nested PCR–RFLP to confirm species identity and exclude natural *L. infantum* infections [32].

### Statistical analysis

Differences between *P. perfiliewi* and *P. perniciosus* were evaluated using *t*-tests (significance *p* ≤ 0.05) for feeding and survival rates, morphotype distribution, parasite loads, and gut localization. The correlation between heavy parasite load and presence of metacyclic promastigotes in *L. major* was assessed using Fisher’s exact test (*F*-test), fixing *p*-value ≤ 0.05 for significant results. All statistical analyses were performed in SPSS.

## Results

### *Phlebotomus perfiliewi* and *Leishmania tropica* experimental infection model

**Fig. 1:**
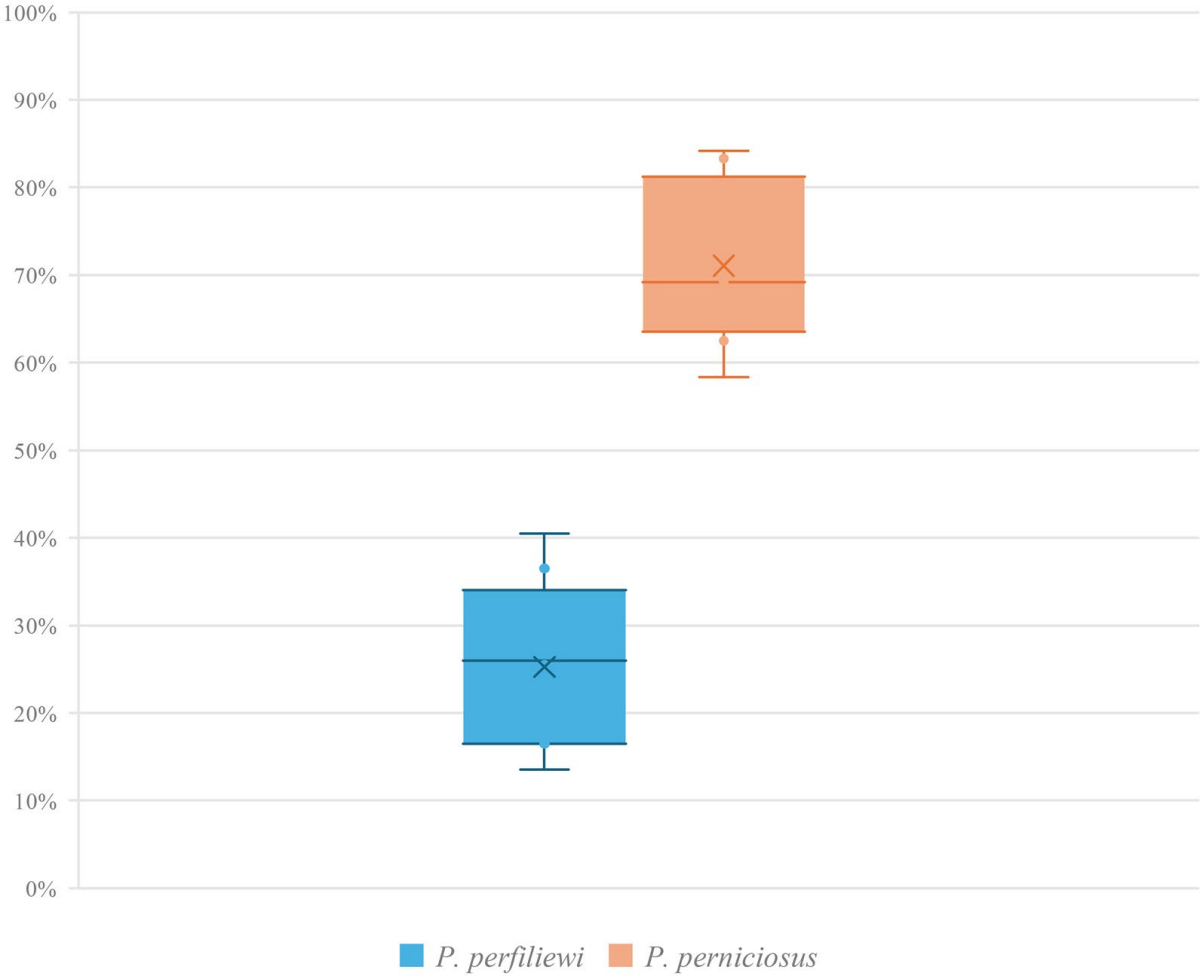
Diagram showing the infection rate of *Leishmania tropica* in *Phlebotomus perfiliewi* and *Ph. perniciosus.* For each species, boxes represent data distribution around the median (horizontal line); “X” indicates the percentage of mean infection rate, and whiskers extend to the minimum and maximum values (whiskers)

A total of 5923 engorged *P. perfiliewi* females were collected across eight experimental replicates (five in 2022 and three in 2023). The mean feeding rate was 25.7% (range: 13.5–40.5%), with no significant difference from the *P. perniciosus* control group (28.5%; *t* = 0.768, df = 13, *P* = 0.456). The average survival rate across replicates was 70.0%. Microscopic analysis revealed an overall *Leishmania* infection prevalence of 25.2% (1492/5923) in *P. perfiliewi*, significantly lower than the 72.8% observed in the *P. perniciosus* control group (83/114; *t* = 17.730, df = 11, *P* < 0.0001) (Fig. 1).

(72.8%; *t* = 17.730, *P* < 0.0001) (Fig. 1). Infection rates peaked at 3 days post blood meal (PBM, 28.8%), declined at days 4–5 (18.2%), and stabilized around 24.6% during days 6–11 PBM.

**Fig. 2:**
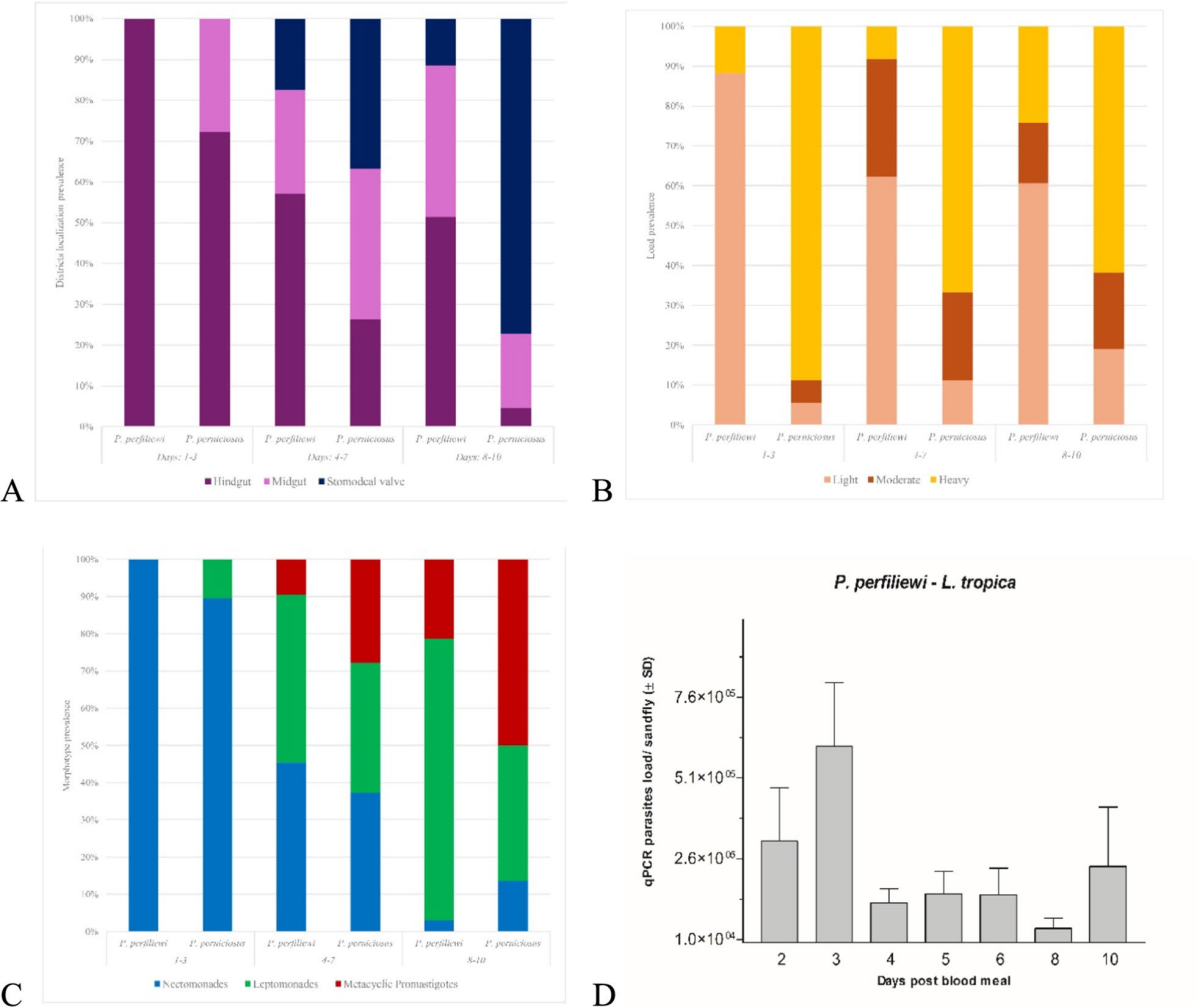
Histograms showing *Leishmania tropica* promastigote infection patterns in *Phlebotomus perfiliewi* and *P. perniciosus* females at different days post-blood meal (DPBM), grouped into three intervals (DPBM: 1–3; 4–7; 8–10): **A** Percentage of infected females showing different promastigote morphotypes (Nectomonads, Leptomonads, Metacyclics); **B** Percentage of infected females with different parasite loads (Light: 1–100; Moderate: 101–1000; Heavy: > 1000 promastigotes); **C** Percentage of infected females with promastigote localization in specific gut regions (hindgut, midgut, stomodeal valve); **D** Mean parasite DNA loads (sand fly/pmol of DNA), as quantified by quantitative real-time PCR (qPCR)

Infection rates varied over time PBM, with the highest prevalence detected at 3 days PBM (28.8%). This was followed by a decline to 18.2% at days 4–5 PBM and stabilization around 24.6% during days 6–11 PBM. Morphological microscopic gut analysis revealed a clear parasite morphotype progression. During the early phase (days 1–3 PBM), 100% of positive females harbored only procyclic and nectomonad promastigotes. From day 4 onward, leptomonads and metacyclic forms began to appear. The prevalence of metacyclic promastigotes increased in the late infection phase (8–11 days PBM), reaching 20.8% in 2022 and 15.8% in 2023 (Fig. 2A). Comparative analysis with *P. perniciosus* showed no significant differences in nectomonad and leptomonad form prevalence. However, *P. perfiliewi* had significantly fewer metacyclic-positive females (*t* = 2.448, df = 12, *P* = 0.031), indicating less efficient metacyclogenesis.

Parasite load followed a similar temporal trend. In early infections (1–3 days PBM), 88.2% of females exhibited light parasite loads. This proportion declined to approximately 62.3% during the mid-phase (4–7 days PBM), and further decreased in the late phase (6–11 days PBM), when heavy infections peaked at 29.6% (2022 data) (Fig. 2B). A significant correlation was observed between heavy parasite load and the presence of metacyclic promastigotes (*F* = 10.231, df = 1, *P* = 0.013). *P. perfiliewi* however supported metacyclogenesis primarily under light parasite loads, unlike *P. perniciosus*, which showed significantly higher overall infection intensities (*t* = 8.203, df = 12, *P* < 0.0001; *t* = 9.479, df = 17, *P* < 0.0001).

Parasite localization within the digestive tract indicated a progressive migration toward the anterior gut. At early stages, 100% of infected females displayed colonization of the midgut and hindgut. Between 4 and 7 days PBM, 42.9% of females showed anterior colonization (cardia/thorax), with values of 38.8% in 2022 and 57.1% in 2023. However, by 8–11 days PBM, only 11.4% of females exhibited colonization of the stomodeal valve (see Additional Figure S2). *P. perniciosus* on the other hand showed significantly higher rates of anterior colonization (*t* = 2.518, df = 13, *P* = 0.026), suggesting more efficient parasite migration toward the valve region (Fig. 2C).

Quantitative PCR analysis conducted on 2023 samples revealed high variability in parasite load, ranging from 1 × 10^−1^ to 3.5 × 10^5^ parasites per gut. These loads appeared independent of both parasite morphotype and viability, reinforcing the findings from microscopic analysis (Fig. 2D).

### *Phlebotomus perfiliewi* and *Leishmania* major experimental infection model:

**Fig. 3:**
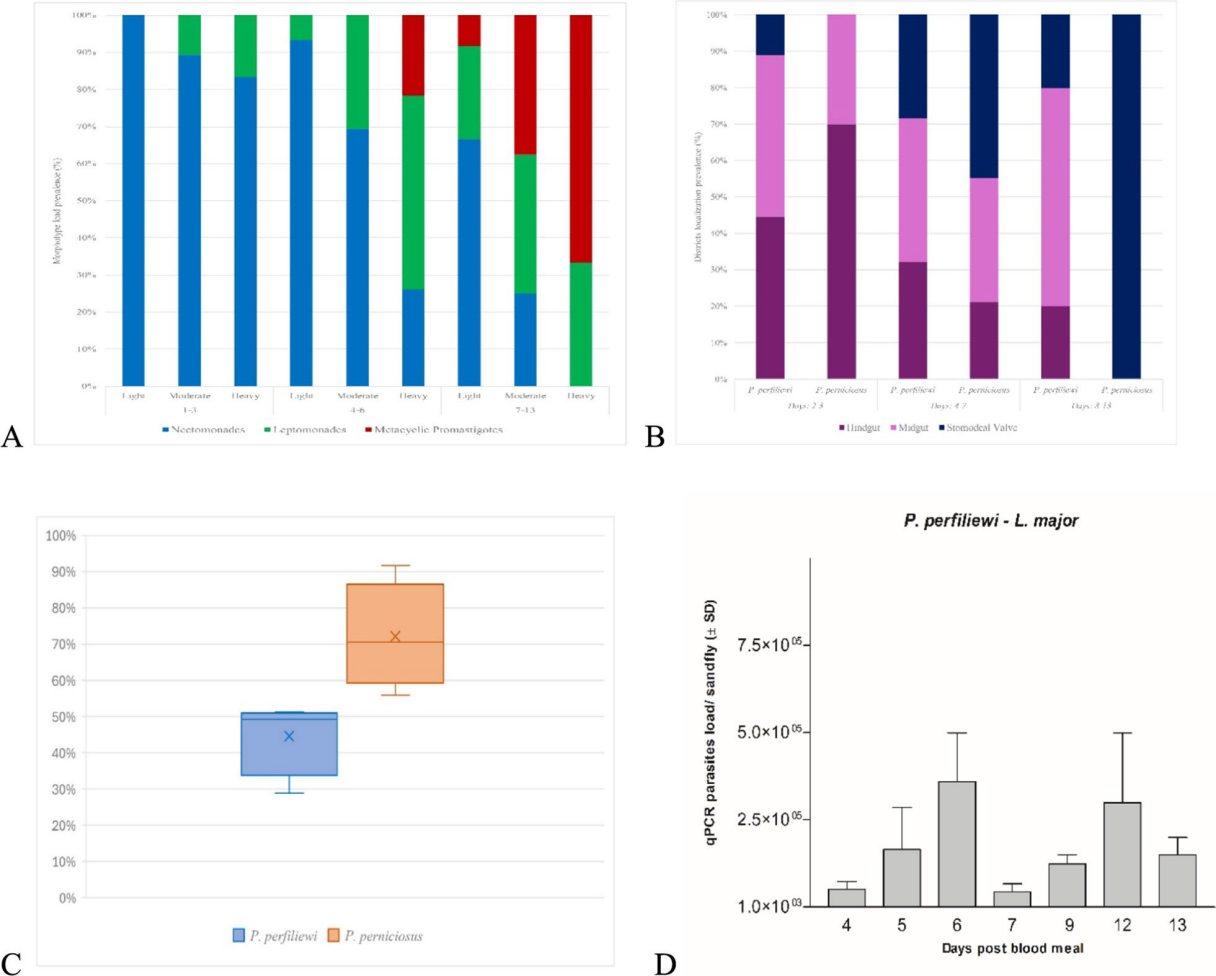
Histograms showing the overall prevalence of *Leishmania major* promastigotes in *Phlebotomus perfiliewi* and *P. perniciosus* females at different post-bloodmeal days (DPBM), grouped into three time categories (DPBM: 1–3; 4–6; 7–13). **A** Parasite load categorized as: Light (1–100), Moderate (101–1000), and Heavy (> 1000), and further classified by promastigote morphotypes: Nectomonads, Leptomonads, and Metacyclics; **B** Localization of promastigotes within the digestive tract: hindgut, midgut, and stomodeal valve; **C** Box plot showing percentage of the infection rate of *L. major*, for each sand fly species, boxes represent data distribution around the median; whiskers show minimum and maximum range, and “X” indicates the mean infection rate; **D** Histograms showing mean parasite DNA loads (sand fly/pmol of DNA), determined by quantitative real-time PCR (qPCR)

From a total of 6333 wild-caught *P. perfiliewi* females across four replicates, a mean feeding rate of 33.1% was observed (range: 8.8–50.2%), significantly lower than that recorded in *the P. perniciosus* control group (56.0%; *t* = 2.435, df = 6, *P* = 0.050). The overall survival rate over the 13-day post-blood meal (PBM) period was 57.7%, with mortality peaks occurring at days 2 and 5 PBM. Microscopic examination revealed an overall *Leishmania* infection rate of 46.6% (145/311), significantly lower than the 70.5% observed in *P. perniciosus* (79/112; *t* = 3.033, df = 5, *P* = 0.030) (Fig. 3C).

Infection prevalence in *P. perfiliewi* remained relatively stable during the first five days PBM (mean = 48.2%, 109/224), followed by peaks at days 6 (59.0%, 23/39) and 13 PBM (50.0%, 2/4). These dynamics were supported by qPCR analysis, which showed parasite loads ranging from 10^5^ to 10⁷ parasites/gut on day 6 and from 10^5^ to 10^6^ on day 13 (Fig. 3D). Morphological stage analysis showed that during the first three days PBM, 53.2% (25/47) of positive females harboured nectomonads, generally at light to moderate parasite loads. Between days 4–6 PBM, a shift in parasite composition was observed. Among 89 positive females, nectomonad infections remained stable (59.6%, *n* = 53), while the number of leptomonad-positive individuals rose to 25.8% (*n* = 23). Metacyclic promastigotes were first detected during this interval in 5.6% of females (*n* = 5). In the late infection phase (7–13 days PBM), metacyclic forms were found at moderate to heavy loads in 21.7% of females (5/23). Earlier stages continued to be present, with 73.9% of females still positive for nectomonads (*n* = 10) and leptomonads (*n* = 7), indicating that metacyclogenesis in *P. perfiliewi* can be achieved under light to moderate parasite loads (*F* = 14.525, df = 1, *P* = 0.007) (Fig. 3A). Comparative analysis revealed a significantly higher prevalence of nectomonads in *P. perfiliewi* compared to *P. perniciosus* (*t* = 3.760, df = 13, *P* = 0.002), while no significant differences were found for leptomonad or metacyclic stages (*P* > 0.1 for both comparisons). Successful anterior migration was evident as regards parasite localization. From days 4 to 13 PBM, promastigotes were most frequently found in the midgut (40.7%, *n* = 35/86) and hindgut (31.4%, *n* = 27/86) (Fig. 11). *P. perfiliewi* showed significantly higher midgut colonization compared to *P. perniciosus* (*t* = 3.235, df = 16, *P* = 0.005), while hindgut colonization rates were comparable (*t* = 0.336, df = 14, *P* = 0.742). By contrast, a significantly greater proportion of *P. perniciosus* females exhibited stomodeal valve colonization (*t* = 2.131, df = 11, *P* = 0.006), suggesting more efficient anterior parasite migration in this control species (Fig. 3B).

## Discussion

In recent years there have been increasing reports of the emergence of non-autochthonous *Leishmania* species in Europe previously considered absent, such as *L. major* and *L. tropica*. According to WHO’s Global Health Observatory Data Repository, estimated cases of CL caused by these species have risen, in part due to the influx of war refugees [4]. Notably, the Syrian civil war has been associated with an increased incidence of *L. tropica* in Syria and neighbouring countries [36], contributing to a broader diagnosis and clinical management of these infections in European healthcare settings [37]. Considering (i) the fact that both *L. tropica* and *L. major* have been molecularly detected in natural *P. perfiliewi* populations [10, 13], and (ii) climate-driven changes in sand fly distribution and density, the aim of this study was to assess whether *P. perfiliewi* is capable of supporting the complete development and, consequently, potential transmission of these parasites. This investigation represents the first experimental infection conducted using a wild-caught *P. perfiliewi* population. The use of a wild population adds to the potential strength of the study of ecological relevance. Unlike laboratory colonies, which may be genetically constrained and poorly adapted to variable environmental conditions, wild-caught flies exhibit greater genetic diversity and resilience. The large number of collected females and their proven opportunistic feeding behaviour [38] offered a sufficient sample size of engorged females, increasing the likelihood of observing late-stage infection and metacyclogenesis, as also reported in reared colonies by Maroli et al. [23]. However, the use of wild populations introduces variability. Newly emerged females for instance may be unwilling to blood-feed due to sexual immaturity, while older females may already be completing a gonotrophic cycle. Even so, sexually mature females demonstrated the ability to survive long enough to allow parasite development, consistent with other studies in which sand flies survived up to 10–16 days post-infection under similar experimental conditions [16, 18, 39]. Intrinsic factors influencing infection success, particularly the interaction between parasite and sand fly midgut, which can critically impact infection outcomes, have been considered. According to the “blocked fly” hypothesis [40], heavy anterior midgut infections may lead to damage of the stomodeal valve, the structure responsible for regulating ingestion and regurgitation during feeding. When damaged, this valve remains open, facilitating high parasite transmission during feeding events or repeated low-dose deliveries resulting in cumulative infective doses. In this study, *P. perfiliewi* showed relatively high infection rates and a notable prevalence of metacyclic forms, especially in *L. major* infection, indicating a possible higher susceptibility to this species rather than to *L. tropica,* under experimental conditions. This is in line with previous findings that indicate variability in vector competence among sand fly species. For example, *P. perniciosus* has demonstrated high infection rates with both *L. tropica* (78%) and *L. major* (85%) [16, 17, 39], comparable to their respective proven vectors, *P. sergenti* (81.5%) and *P. papatasi* (85%) [41–43]. Interestingly, *P. perfiliewi* exhibited higher infection prevalence with *L. tropica* than reported for *P. papatasi* (11%) suggesting that it may support parasite development more efficiently under laboratory conditions [42]. These findings underline the adaptability of *L. tropica* and *L. major* to different sand fly species, even in sympatric wild populations, reflecting a plasticity that may facilitate their spread, similar to the observed expansion of *L. infantum* in the New World [43]. The current geographic distribution of *P. perfiliewi*, also covering several Middle Eastern countries [4], raises epidemiological concerns regarding its potential expansion into areas endemic for *L. major* and *L. tropica* due to ongoing climate change. The demonstrated ability of *P. perfiliewi* to support the development of both *Leishmania* species under experimental conditions suggests a possible, though unconfirmed, role in their transmission, highlighting the importance of strengthened entomological surveillance to monitor its potential spread into new areas.

As Di Muccio et al. [44] point out, outbreaks of exotic *Leishmania* species in Europe remain unlikely under current conditions, largely due to strong public health surveillance systems and unfavourable environmental factors. However, the potential for non-autochthonous *Leishmania* species to adapt to local reservoirs and vectors cannot be entirely excluded. This possibility underscores the importance of integrated surveillance strategies that monitor not only vector populations but also domestic and wild reservoir hosts, as well as seasonal dynamics and the geographic expansion of native sand fly vector species. One clear limitation of this work is the lack of in vivo confirmation of parasite transmissibility to a susceptible host. Future studies should therefore include xenodiagnostic assays to evaluate the actual transmission potential of infected sand fly populations. Finally, the opportunistic feeding behaviour of *P. perfiliewi*, which includes a broad range of wild animals [22, 31], together with its apparent permissiveness to *Leishmania* infections, could potentially facilitate the introduction and establishment of non-endemic parasite species under suitable ecological conditions.

## Conclusions

In some specific epidemiological contexts, the presence of permissive vector species could be hypothesized to lead to an increase in the possible spread of exotic *Leishmania* species. In this study, the *P. perfiliewi* permissive attitude was evaluated by means of experimental infections with *L. tropica* and *L. major*.

Although this sand fly species appears able to support the developmental cycle of both parasites, it has been shown to have a higher susceptibility to *L. tropica*, as indicated by the greater metacyclic load in the stomodeal valve compared with *L. major*. These differences may reflect distinct epidemiological scenarios. In Italy, the presence of a permissive vector species could be sufficient to sustain the life cycle and local transmission of anthroponotic species such as *L. tropica*. In contrast, zoonotic *L. major* requires more specific eco-epidemiologic conditions, including the presence of susceptible vertebrate hosts, which may limit its establishment in Italian environments. It should be noted, however, that these results are based only on laboratory infections and lack in vivo confirmation or xenodiagnosis, therefore do not demonstrate the ability of *P. perfiliewi* to sustain natural transmission. Nevertheless, ongoing climate changes and human-driven factors such as travel, migration, trading of animals and environmental modifications could create favourable conditions for the introduction and adaptation of these two *Leishmania* species, in areas where *P. perfiliewi* is prevalent. The implementation of integrated surveillance strategies within a One Health approach would therefore be advisable to monitor potential changes in the epidemiology of exotic and endemic *Leishmania* species.

## Supplementary Information

Supplementary Material 1.

Supplementary Material 2.

## Data Availability

All the data are included within the article and its additional files.

## Abbreviations

WHO: World Health Organization
VL: Visceral leishmaniasis
CL: Cutaneous leishmaniasis
PBS: Phosphate-buffered saline
qPCR: Quantitative PCR
PBM: Post blood meal
RFLP: Restriction Fragment Length Polymorphism
