# Supplementary figures and images for: Evaluation of an Italian Phlebotomus perfiliewi (Larroussius) wild population as a permissive vector species for Leishmania tropica and L. major transmission
Source: Parasit Vectors. 2025 Dec 5;19:19. doi: 10.1186/s13071-025-07170-7 (PMC12797989; doi:10.1186/s13071-025-07170-7)

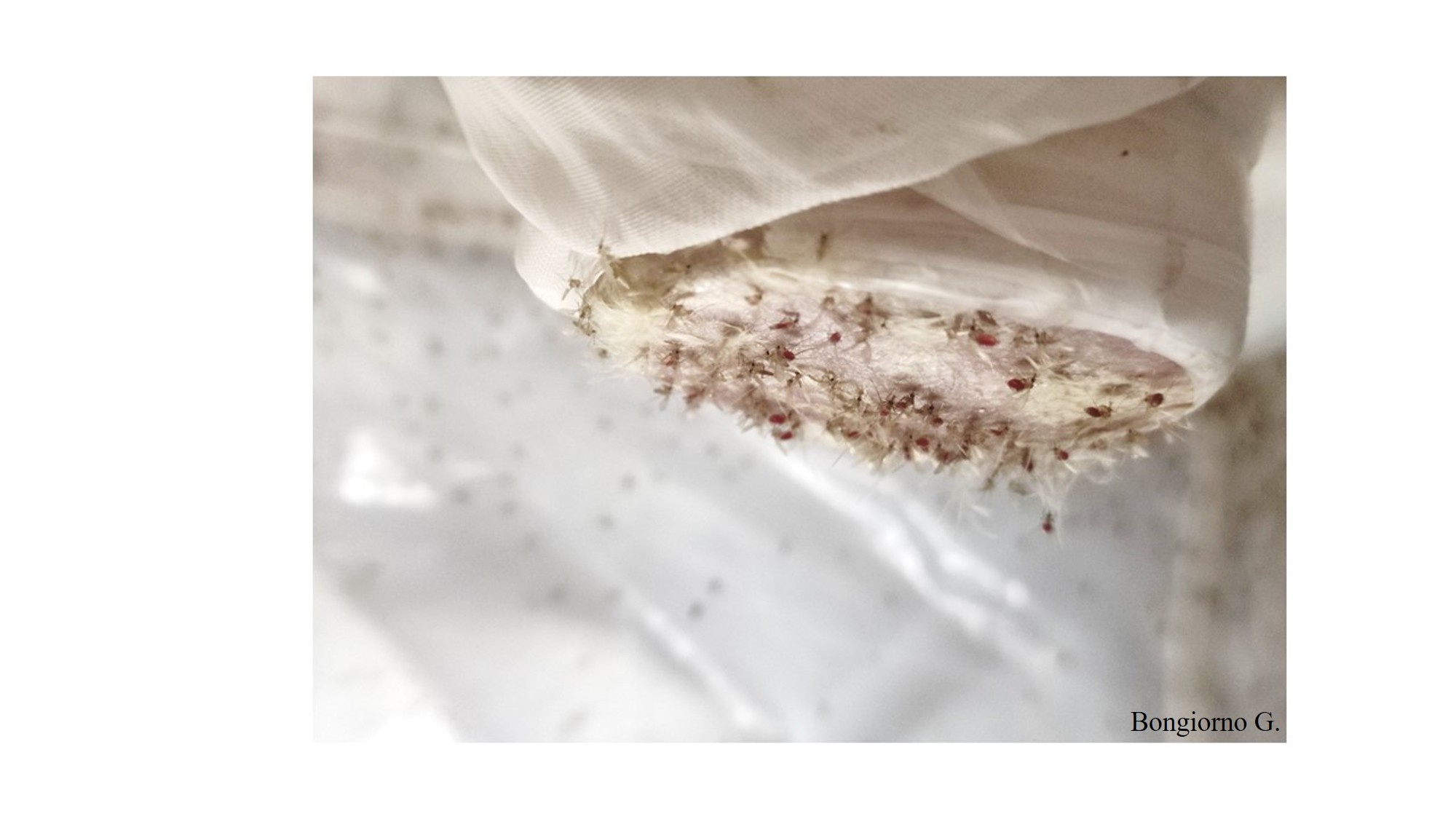

Supplement: Supplementary file 1 — Supplementary Material 1. [file 13071_2025_7170_MOESM1_ESM.jpg]

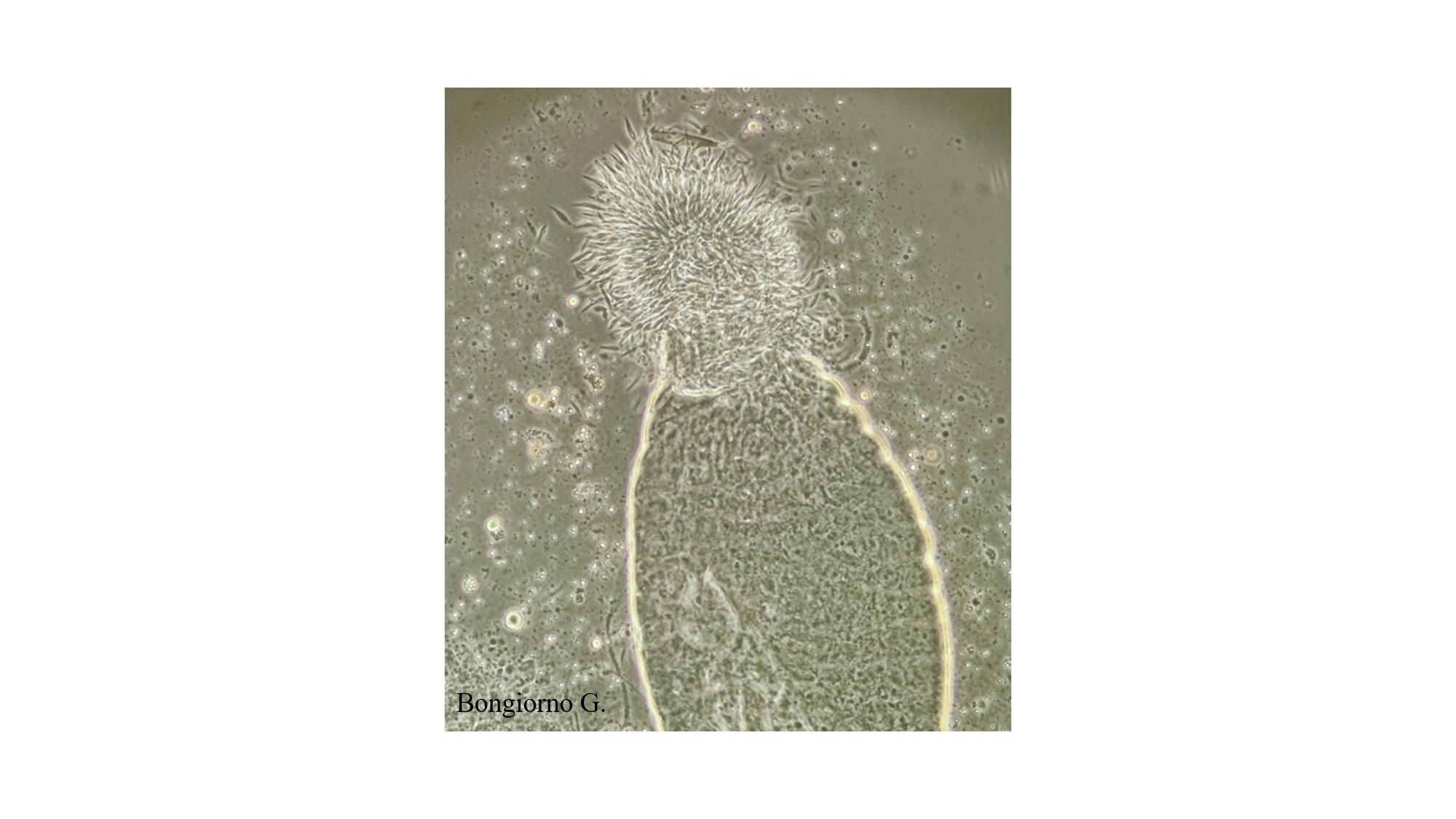

Supplement: Supplementary file 2 — Supplementary Material 2. [file 13071_2025_7170_MOESM2_ESM.jpg]
